# BiGG Models 2020: multi-strain genome-scale models and expansion across the phylogenetic tree

**DOI:** 10.1093/nar/gkz1054

**Published:** 2019-11-07

**Authors:** Charles J Norsigian, Neha Pusarla, John Luke McConn, James T Yurkovich, Andreas Dräger, Bernhard O Palsson, Zachary King

**Affiliations:** 1 Department of Bioengineering, University of California, San Diego, La Jolla, CA 92093, USA; 2 Institute for Systems Biology, Seattle, WA 98109, USA; 3 Computational Systems Biology of Infection and Antimicrobial-Resistant Pathogens, Institute for Biomedical Informatics (IBMI), University of Tübingen, 72076 Tübingen, Germany; 4 Department of Computer Science, University of Tübingen, 72076 Tübingen, Germany; 5 German Center for Infection Research (DZIF), 72076 Tübingen, Germany; 6 Department of Pediatrics, University of California, San Diego, La Jolla, CA 92093, USA; 7 Novo Nordisk Foundation Center for Biosustainability, Technical University of Denmark, Kemitorvet, Building 220, 2800 Kongens Lyngby, Denmark

## Abstract

The BiGG Models knowledge base (http://bigg.ucsd.edu) is a centralized repository for high-quality genome-scale metabolic models. For the past 12 years, the website has allowed users to browse and search metabolic models. Within this update, we detail new content and features in the repository, continuing the original effort to connect each model to genome annotations and external databases as well as standardization of reactions and metabolites. We describe the addition of 31 new models that expand the portion of the phylogenetic tree covered by BiGG Models. We also describe new functionality for hosting multi-strain models, which have proven to be insightful in a variety of studies centered on comparisons of related strains. Finally, the models in the knowledge base have been benchmarked using Memote, a new community-developed validator for genome-scale models to demonstrate the improving quality and transparency of model content in BiGG Models.

## INTRODUCTION

BiGG Models (http://bigg.ucsd.edu) was initially released in 2010 as a knowledge base of biochemically, genetically and genomically structured genome-scale metabolic network reconstructions, and the first release was followed by a complete redesign in 2016 (1,2). Since its initial release, the BiGG Models publications have been cited over 450 times (via Web of Science) and the website maintains a user base of ∼2000 monthly active users. BiGG Models is built around a workflow for standardizing models that is meant to verify and, in some cases, improve model quality. External studies have also indicated the high quality of models in BiGG. In one instance, the robustness of growth predictions for models in BiGG was demonstrated and used as a benchmark for a new collection of microbiome metabolic models (3). Another study on ‘erroneous energy generating cycles’—a common issue in metabolic models—found that models in BiGG were less likely to have these undesirable cycles than models from other databases (4). A number of projects have used BiGG to automate reconstruction workflows and analyses (5–7).

With the BiGG Models 2020 update, we have included an additional 31 genome-scale metabolic models (GEMs) across four independent releases (versions 1.3–1.6), introduced the ability to download sets of multi-strain models that have been generated from a given base reconstruction page and continuously improved features with suggestions and contributions from the open source community. New content has increased the utility of the knowledge base for the community by expanding the number of organisms and metabolic processes represented. The BiGG Models architecture has been designed to enable these advances and continually improve the knowledge base.

## KNOWLEDGE BASE CONTENT

BiGG Models continues to contain high-quality, manually curated GEMs collected from various publications. Quality control in BiGG Models begins with our requirement that all models undergo rigorous peer review before entry. We begin our import workflow with the exact model that was reported in a peer-reviewed publication, and the workflow is designed to improve the quality of annotations and standardization in the model, without making any changes to the reaction content, parameterization or relationships (e.g. gene–reaction rules).

To load a model into BiGG, first each model is aligned to the shared namespace of reactions and metabolites across all models. When identifiers can be improved automatically (e.g. by finding a universal reaction based on the reactants), the workflow does this automatically; in other cases, non-matching identifiers are left as is to ensure that model content does not change. Next, genome annotations are loaded into the database for each model, providing explicit links between metabolic reactions and genes. When adding content to the BiGG Models database, manual efforts are made to ensure that each metabolite identifier follows the specified naming convention, each reaction contains a unique identifier and gene–reaction rules are properly represented in valid Boolean logic. When obvious errors are identified (typos, duplicate metabolites), these are corrected manually, with feedback from the model authors. The coalescence of genome annotation information, with external database links, and reaction, metabolite, and gene information from peer-reviewed models drives the quality of the knowledge base.

To ensure that model content (the reaction connectivity, gene–reaction rules and parameters that affect model predictions) has not changed from the peer-reviewed version presented in the original publication, an internal testing suite runs 18 tests for each model, for a total of >1900 tests. For example, tests ensure that reaction, metabolite, and gene counts have not changed, that all reactions that were mass balanced in the published model are still balanced and that genes have mapped to genome annotations correctly. An additional 36 tests are included to spot-check bugs and edge cases that have appeared during previous builds of BiGG Models. The full test suite is available in the source code (https://github.com/SBRG/bigg_models/blob/master/bigg_models/tests).

**Figure 1.**
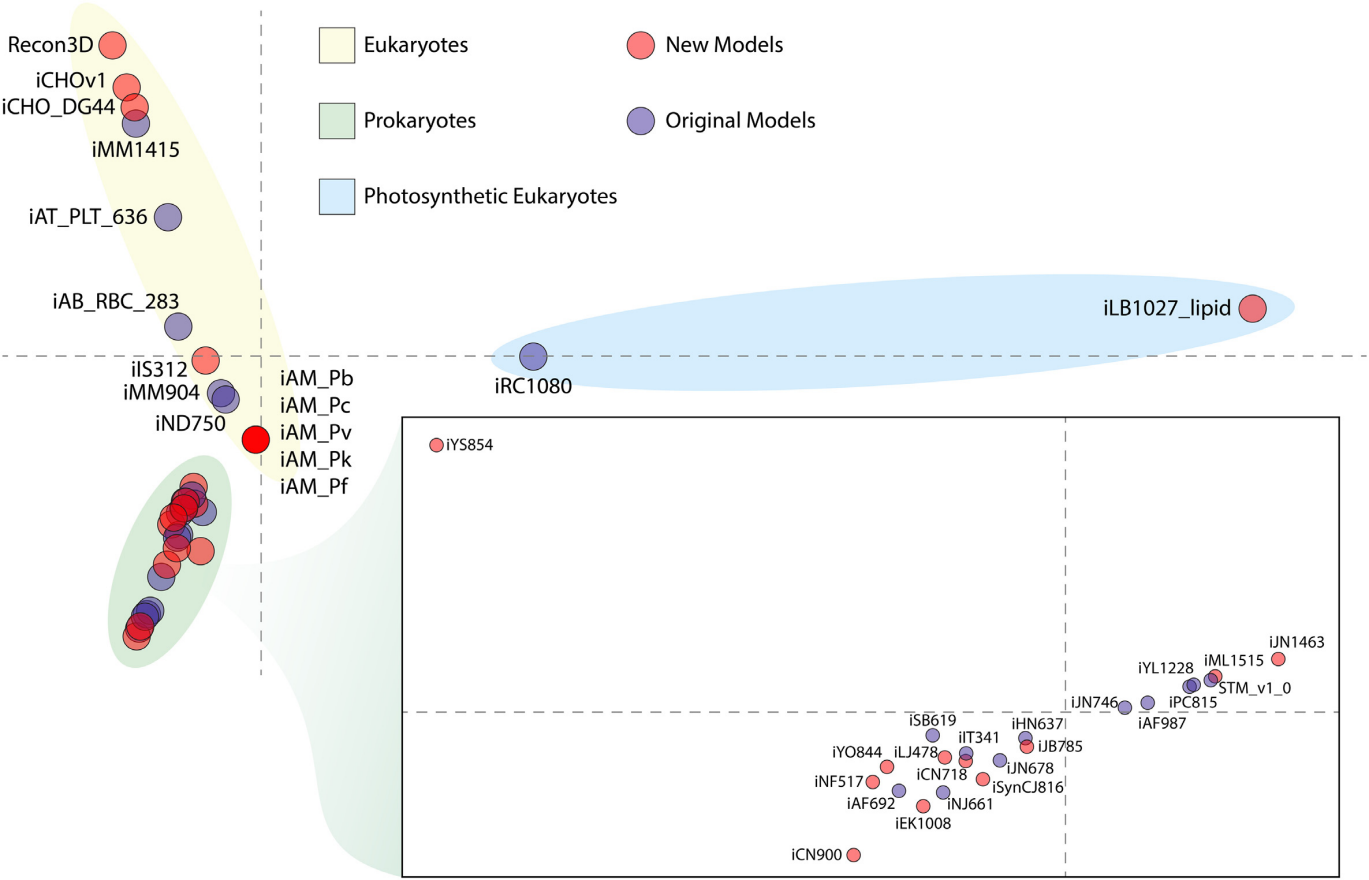
Multiple correspondence analysis of the reaction presence or absence within each model clusters models according to eukaryotic (yellow ellipse), prokaryotic (green ellipse and inset) and photosynthetic eukaryotes (blue ellipse) within metabolic reaction space. Dimension 1 (*x*-axis) explained 14.5% of the variance; dimension 2 (*y*-axis) explained 14.2%. Further, a number of the models newly introduced within this update (red circles) are found at edges of the MCA plot, indicating that within these two dimensions, they contribute to additional diversity in reaction content compared to the previous release. For this analysis, iML1515 was used as a representative *E. coli* model and iIS312 as representative for *Trypanosoma cruzi*.

In the 2016 release of BiGG Models, there were 77 GEMs; with this update, we detail 31 additional models, covering release versions 1.3–1.6 (http://bigg.ucsd.edu/updates), and bringing the total to 108 GEMs (8–13). Genome annotations for each model (where possible) are downloaded from the National Center for Biotechnology reference sequence database (14) and linked to the corresponding GEM. Notable additions are the Recon3D, iCHOv1 and iML1515 (15–17) for the human metabolic network, Chinese hamster ovary cell and *Escherichia coli* K-12 MG1655, respectively. BiGG Models continues to host gold-standard models within a shared knowledge base of biological reactions and metabolites. We also demonstrate that the new GEMs valuably expand the portion of the reactome encapsulated by the knowledge base. The number of unique reactions represented in the database more than doubled from 11,459 in the 2016 version to 28,302. Likewise, the number of unique metabolites has more than doubled from 4,040 to 9,088. In addition to expanding the number of metabolic processes within the database, we sought to evaluate the diversity of reaction presence among GEMs within the database. Reaction presence or absence of the shared namespace was identified for every representative GEM, and this matrix was subject to multiple correspondence analysis (Figure 1). Notably, this analysis shows that new models within the update exist at the edge of each cluster demonstrating that the new content is increasing the level of dissimilarity among GEM reaction content. This separation among models conveys that the metabolic space within BiGG Models is moving past representations of shared common pathways and incorporating an increasing amount of organism-specific biochemical capabilities.

This update also includes multi-strain models, a recent development within the metabolic modeling community. We define multi-strain models as those generated via the ability to extend the content contained within a gold-standard reconstruction to related strains of interest. This technique has proven insightful in a number of studies for comparative analysis of strains (18–24). Thus, we have included a means for the hosting of the draft strain-specific models generated within these studies on BiGG Models. Each strain-specific model is available to download within a zip folder from the page of the base reconstruction used to generate the strain-specific models. The GEMs of iCN718, iYL1228 and STM_v1_0 (18,25,26) each contain datasets of multi-strain models linked from their reconstruction pages within BiGG Models. Identifiers in multi-strain reconstructions are inherently BiGG Models compliant as they have been generated through the use of a hosted model. These multi-strain models have demonstrated value in comparative simulation to identify key differences among the strains of a species and they all represent starting points toward manually curated reconstructions for each strain should the proper steps be undertaken (27).

## VALIDATION OF MODELS WITH MEMOTE

BiGG Models now links to the model validation tool, Memote, which evaluates and scores GEMs with a set of community-maintained tests (28). Consistent with the efforts in BiGG Models to maximize the value of metabolic models, evaluation with Memote provides a means to quantify model quality. Quality, in this case, indicates that GEMs adhere to established standards such as consistent identification of model components and biologically feasible results under varied growth conditions. This standardized approach to model validation ensures the quality of BiGG Models content and provides a benchmark for continued improvement.

**Figure 2.**
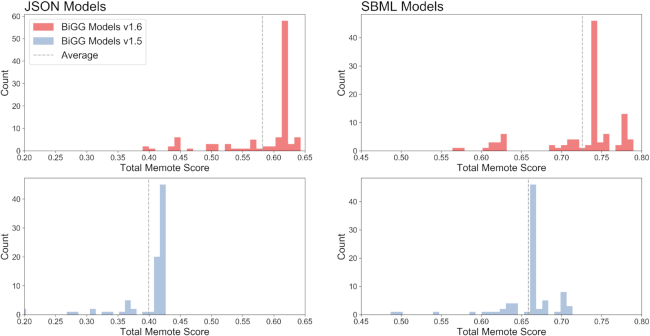
The latest update has resulted in improved Memote annotation scores for both JSON and SBML model formats. See Supplementary Table S1 for detailed score information for each model.

Both the original 77 GEMs included in the 2016 release of BiGG and the 31 GEMs included in this update were evaluated with Memote (Figure 2). Largely due to improved gene, metabolite, and reaction annotations, the average Memote score of JSON-formatted models increased from 40% to 58%, while that of the SBML-formatted (29–31) models advanced from 66% to 73% (Supplementary Table S1). While these scores represent significant improvements, ongoing database annotation efforts will be necessary to maximize Memote scores for models in BiGG. Memote does not currently support testing of MATLAB-formatted models; however, BiGG generates MATLAB-formatted models using the same data sources as the JSON-formatted files, so equivalent model content is present. These results highlight the value of BiGG Models as a knowledge base of GEMs, and scoring its content with Memote reinforces its effort to provide access to GEMs with thorough and consistent standards.

## ADDITIONAL FEATURES AND IMPROVEMENTS

Regular improvements are made to BiGG Models that have made the knowledge base faster, easier to use and better for analysis. Filters are now provided during search to filter out multi-strain reconstructions in the search results (see the toggle titled ‘Exclude multi-strain models from search’). Gene and protein sequences are now included directly in the database and available by API. A new advanced search feature allows users to identify all gene and protein sequences for any universal BiGG reaction (see ‘Find sequences for BiGG Models reaction’ on the advanced search page).

A new ‘universal’ model was added for download on the Data Access page; this model provides all reactions and metabolites from BiGG in a single COBRA-compatible JSON file, so users can rapidly add BiGG content to their own computational workflows using COBRA tools. Namespace downloads on the Data Access page have also been extended to include old and deprecated identifiers. External database links are regularly updated with the latest information from MetaNetX (32). Many manual improvements have been made to annotations, including better gene mapping for yeast models. SBML downloads have improved through regular updates to the ModelPolisher project (https://github.com/draeger-lab/ModelPolisher).

Since the 2016 release of BiGG Models, the website has been deployed on a new server to dramatically improve speed when searching and browsing. Finally, bugs and suggestions are collected on GitHub (https://github.com/SBRG/bigg_models), and this has led to continuous and transparent improvements to the site by the BiGG Models team.

## CONCLUSION

BiGG Models continues to be a widely used and well-maintained platform for integrating, sharing and standardizing GEMs. The updated knowledge base integrates the metabolic knowledge for 108 GEMs, as well as including the content for 515 draft strain-specific models across three organisms, all available within the knowledge base. BiGG Models is free for academic use and continues to extend the content within the knowledge base. Further, all source code continues to be available on GitHub to enable submission of potential bugs. The development of BiGG Models continues to evolve with the needs of the research community, introducing multi-strain models and validation through Memote testing. Future BiGG Models releases will continue to be shaped by the feedback from users.

## DATA AVAILABILITY AND REQUIREMENTS

BiGG Models is freely available online for academic and non-profit use at http://bigg.ucsd.edu, under the BiGG License described at http://bigg.ucsd.edu/license. While the content of BiGG is restricted to academic and non-profit use to protect intellectual property claims, the source code is open source and available to all users under the MIT license at https://github.com/SBRG/bigg_models. Installation of an independent system requires Python 3.5 and PostgreSQL 9.4 or later.

We encourage community members to submit their model content to BiGG Models, and the website includes a section that describes the minimum requirements for inclusion in BiGG and the process for submitting a new model: http://bigg.ucsd.edu/about These requirements reflect the quality standards set by BiGG Models: identifier standardization for reactions and metabolites, links to genome annotations and peer-reviewed publication as the primary means of verifying model quality.

## Supplementary Material

gkz1054_Supplemental_Files
