# Supplementary material for: BiGG Models 2020: multi-strain genome-scale models and expansion across the phylogenetic tree
Source: Nucleic Acids Res. 2019 Nov 7;48(D1):D402–6. doi: 10.1093/nar/gkz1054 (PMC7145653; doi:10.1093/nar/gkz1054)
Supplement: gkz1054_Supplemental_Files [file gkz1054_supplemental_files.zip › Supplementary legend.pdf]

Supplementary legend

Supplementary Table 1: Memote scores for the following tests: consistency, total_score, annotation_met, annotation_rxn, annotation_gene, and annotation_sbo. A score for each metric is reported for both the JSON and SBML versions of models within v1.5 and v1.6.
