# Supplementary material for: BiGG Models 2020: multi-strain genome-scale models and expansion across the phylogenetic tree
Source: Nucleic Acids Res. 2019 Nov 7;48(D1):D402–6. doi: 10.1093/nar/gkz1054 (PMC7145653; doi:10.1093/nar/gkz1054)
Supplement: gkz1054_Supplemental_Files [file gkz1054_supplemental_files.zip › SupplementaryTable1.pdf]

Models	Test	BiGG Models v1.6 JSON Tests Performance	BiGG Models v1.5 JSON Tests Performance	BiGG Models v1.6 SBML Tests Performance	BiGG Models v1.5 SBML Tests Performance
e_coli_core	consistency	0.9971	0.854227405	0.9970845481049563	0.9970845481049563
	total_score	0.6431	0.373135219	0.730308885776783	0.653402611339348
	annotation_met	0.7254	0.25	0.8162878787878788	0.8147095959595959
	annotation_rxn	0.7418	0.302631579	0.8339181286549708	0.7742690058479532
	annotation_gene	0.6324	0	0.632360097323601	0.6630170316301703
	annotation_sbo	0.2737	0	0.45454545454545453	0.2727272727272727
iAB_RBC_283	consistency	0.8878	0.702907712	0.9144907233402808	0.8457648546144121
	total_score	0.5542	0.313390905	0.7253982698693413	0.6196802789018192
	annotation_met	0.6979	0.25	0.788809144072302	0.7866826156299841
	annotation_rxn	0.7149	0.290511727	0.8024164889836531	0.7553304904051172
	annotation_gene	0.5441	0	0.5417624521072797	0.5112068965517241
	annotation_sbo	0.1818	0	0.5444096133751306	0.36363636363636365
iAF1260	consistency	0.9815	0.981282686	0.9814258399344442	0.9810637705374547
	total_score	0.6334	0.421010375	0.7580318303677548	0.6817039270094232
	annotation_met	0.7024	0.25	0.793342598648354	0.7916802921299324
	annotation_rxn	0.7206	0.281905961	0.8126807538016606	0.7610551357402743
	annotation_gene	0.6332	0	0.6332275971451229	0.666243721913825
	annotation_sbo	0.2728	0	0.5454545454545454	0.36363636363636365
iAF1260b	consistency	0.9815	0.980914959	0.9813186813186814	0.9809149593039573
	total_score	0.6334	0.420861432	0.7579888314610207	0.6816405518509173
	annotation_met	0.7024	0.25	0.793342598648354	0.7916802921299324
	annotation_rxn	0.7206	0.281825796	0.8126807538016606	0.7609808300763075
	annotation_gene	0.6332	0	0.6332275971451229	0.666243721913825
	annotation_sbo	0.2728	0	0.5454545454545454	0.36363636363636365
iAF692	consistency	0.8477	0.846062161	0.988919303432299	0.988919303432299
	total_score	0.5679	0.367939901	0.7107851793087491	0.6335547977055102
	annotation_met	0.7065	0.25	0.7973726114649681	0.7946945570353213
	annotation_rxn	0.7347	0.275362319	0.8314009661835748	0.7760064412238326
	annotation_gene	0.3667	0	0.36666666666666664	0.3872832369942197
	annotation_sbo	0.2729	0	0.45454545454545453	0.2727272727272727
iAM_Pb448	consistency	0.9802		0.980155403464001	
	total_score	0.6169		0.7817351743044506	

	annotation_met	0.6273		0.7741115473673613	
	annotation_rxn	0.7051		0.7843902946995731	
	annotation_gene	0.4655		0.46547619047619043	
	annotation_sbo	0.2728		0.6363636363636364	
iAM_Pc455	consistency	0.9803		0.980298714824827	
	total_score	0.6133		0.7781526332372393	
	annotation_met	0.6276		0.7741806154154556	
	annotation_rxn	0.7025		0.784476515621767	
	annotation_gene	0.3998		0.3997802197802198	
	annotation_sbo	0.2728		0.6363636363636364	
iAM_Pf480	consistency	0.9804		0.9803953512593127	
	total_score	0.6169		0.7817266224103375	
	annotation_met	0.6277		0.774077407740774	
	annotation_rxn	0.7051		0.784343900687391	
	annotation_gene	0.4637		0.46374999999999994	
	annotation_sbo	0.2728		0.6363636363636364	
iAM_Pk459	consistency	0.9803		0.9802468662000547	
	total_score	0.6096		0.7744291248514017	
	annotation_met	0.6277		0.774077407740774	
	annotation_rxn	0.7051		0.7844197302028628	
	annotation_gene	0.3333		0.3333333333333333	
	annotation_sbo	0.2728		0.6363636363636364	
iAM_Pv461	consistency	0.9804		0.9803938376310579	
	total_score	0.6133		0.7781348007622924	
	annotation_met	0.6277		0.774077407740774	
	annotation_rxn	0.705		0.7842712842712842	
	annotation_gene	0.3992		0.3992046276211136	
	annotation_sbo	0.2728		0.6363636363636364	
iAPECO1_1312	consistency	0.9678	0.967760815	0.9695297545724154	0.9695297545724154
	total_score	0.6128	0.416029131	0.7379706500112164	0.6613009016797601
	annotation_met	0.6994	0.25	0.7902701057953375	0.7888423368598446
	annotation_rxn	0.7167	0.285648995	0.8073024578509039	0.7574852732073938
	annotation_gene	0.3667	0	0.3666666666666667	0.3882711348057883
	annotation_sbo	0.2728	0	0.5454545454545454	0.36363636363636365

iAT_PLT_636	consistency	0.8115	0.624007937	0.8283886741468478	0.7668650793650793
	total_score	0.5231	0.28148883	0.6911286702994154	0.5900511971857199
	annotation_met	0.6853	0.25	0.7761763981276176	0.7747905888149791
	annotation_rxn	0.7032	0.27405754	0.7919697971781305	0.7502755731922399
	annotation_gene	0.5507	0	0.550733752620545	0.5527777777777777
	annotation_sbo	0.1818	0	0.5454545454545454	0.36363636363636365
iB21_1397	consistency	0.9722	0.9722	0.9722	0.9722
	total_score	0.6182	0.4178	0.7427	0.6665
	annotation_met	0.6994	0.25	0.7903	0.7889
	annotation_rxn	0.7169	0.2865	0.8076	0.758
	annotation_gene	0.4327	0	0.4327	0.4619
	annotation_sbo	0.2728	0	0.5455	0.3636
iBWG_1329	consistency	0.971362989552303	0.97136299	0.9716063577684143	0.9716063577684143
	total_score	0.614173033333217	0.417424197	0.738798348142427	0.6626430927526883
	annotation_met	0.699484584169037	0.25	0.7903936750781286	0.7889593731050888
	annotation_rxn	0.716830840325915	0.28557096	0.8074506465604605	0.7576513032550976
	annotation_gene	0.366666666666666	0	0.3666666666666667	0.3975169300225734
	annotation_sbo	0.272793605518888	0	0.5454545454545454	0.36363636363636365
iCHOv1	consistency	0.925794132651596	0.925794133	0.9257941326515967	0.9257941326515967
	total_score	0.595523330613620	0.398832948	0.756807463373732	0.6815969104501661
	annotation_met	0.666522156030683	0.25	0.7574312469397748	0.7560439448343398
	annotation_rxn	0.687799122850901	0.273825604	0.7743759067487118	0.7390022845898577
	annotation_gene	0.432899207248018	0	0.43289920724801817	0.46145715364288414
	annotation_sbo	0.272754560462800	0	0.6363636363636364	0.45454545454545453
iCHOv1_DG44	consistency	0.926488414613961		0.926488414613961	
	total_score	0.596291918973985		0.7574460880203691	
	annotation_met	0.671491358514259		0.7624004494233502	
	annotation_rxn	0.689469530413213		0.7743953999661762	
	annotation_gene	0.432882882882882		0.43288288288288285	
	annotation_sbo	0.272773396061067		0.6363636363636364	
iCN718	consistency	0.398892969539452		0.5362736437815762	
	total_score	0.389582290427570		0.6060831178151298	
	annotation_met	0.684275184275184		0.7951474201474201	
	annotation_rxn	0.730405035577449		0.8215380405035577	
	annotation_gene	0.333333333333333		0.3333333333333333	

	annotation_sbo	0.272816838334079		0.6363636363636364	
ICN900	consistency	0.589813571104552			
	total_score	0.462707095142438			
	annotation_met	0.637570621468926			
	annotation_rxn	0.718741524274477			
	annotation_gene	0.394037037037037			
	annotation_sbo	0.272397202291425			
ic_1306	consistency	0.971464982748986	0.971464983	0.9717099156985919	0.9717099156985919
	total_score	0.614177201700084	0.417478359	0.7386531533759837	0.662329512140965
	annotation_met	0.699297990232907	0.25	0.7902070811419984	0.788798365890308
	annotation_rxn	0.716536235428385	0.285766691	0.8070432868672046	0.757265427569903
	annotation_gene	0.366666666666666	0	0.3661573720397249	0.3918767507002801
	annotation_sbo	0.272793970519575	0	0.5451767483853046	0.36363636363636365
iE2348C_1286	consistency	0.970834198365281	0.970926713	0.9712052558791965	0.9712977709185042
	total_score	0.612154450438909	0.417291576	0.7368221667373244	0.6404589309241243
	annotation_met	0.700080534369226	0.25	0.7909896252783173	0.789544743948079
	annotation_rxn	0.716755045833847	0.286071032	0.8072820323097792	0.7573375262054507
	annotation_gene	0.333333333333333	0	0.3333333333333333	0.0
	annotation_sbo	0.272794538055359	0	0.5454545454545454	0.36363636363636365
iEC042_1314	consistency	0.974748315592072	0.974790423	0.9749135224964609	0.9749135224964609
	total_score	0.619199805660449	0.4187833	0.7437930017682396	0.667670497698385
	annotation_met	0.699789955631077	0.25	0.790699046540168	0.7892830170867554
	annotation_rxn	0.716838614591009	0.285924834	0.8074387947269304	0.7576660116269549
	annotation_gene	0.433028919330289	0	0.43302891933028914	0.46440892947742257
	annotation_sbo	0.272794265425068	0	0.5454545454545454	0.36363636363636365
iEC1344_C	consistency	0.546329114455133		0.6891862573122758	
	total_score	0.448374762788441		0.6288506927292888	
	annotation_met	0.692194697753125		0.7887444768261728	
	annotation_rxn	0.717269911143719		0.8075120241297791	
	annotation_gene	0.366666666666666		0.3666666666666667	
	annotation_sbo	0.272793970519575		0.5454545454545454	
iEC1349_Crooks	consistency	0.546395668444343		0.6892528113014865	
	total_score	0.448344508564469		0.6288084155339644	
	annotation_met	0.692072316173035		0.7884705222834719	

	annotation_rxn	0.716638445412030		0.8068658280922432	
	annotation_gene	0.366666666666666		0.3666666666666667	
	annotation_sbo	0.272793244491357		0.5454545454545454	
iEC1356_Bl21DE3	consistency	0.538188972605305		0.6810461154624482	
	total_score	0.445157710853275		0.625613340802012	
	annotation_met	0.692186463171864		0.7885463077068916	
	annotation_rxn	0.716585563665855		0.8067416869424169	
	annotation_gene	0.366666666666666		0.3666666666666667	
	annotation_sbo	0.272793629727936		0.5454545454545454	
iEC1364_W	consistency	0.533276997066355		0.6761341399234981	
	total_score	0.443271597697649		0.6237024234596028	
	annotation_met	0.692656979761286		0.7886375430485446	
	annotation_rxn	0.716443560057887		0.8066409390577263	
	annotation_gene	0.366666666666666		0.3666666666666667	
	annotation_sbo	0.272793053545586		0.5454545454545454	
iEC1368_DH5a	consistency	0.539564953620193		0.6824220964773361	
	total_score	0.443811011528637		0.6242836842433701	
	annotation_met	0.691778109128186		0.7882787381762266	
	annotation_rxn	0.716594698332733		0.8068349926032545	
	annotation_gene	0.333333333333333		0.3333333333333333	
	annotation_sbo	0.272792698485393		0.5454545454545454	
iEC1372_W3110	consistency	0.536719874333061		0.6795770171902048	
	total_score	0.444708051934033		0.625092929508301	
	annotation_met	0.693572850507157		0.7887477486017632	
	annotation_rxn	0.716843928772862		0.8072274595117235	
	annotation_gene	0.366666666666666		0.36666666666666664	
	annotation_sbo	0.272793196651064		0.5454545454545454	
iEC55989_1330	consistency	0.970172217469789	0.970172217	0.9706558641658779	0.9706558641658779
	total_score	0.616916532609332	0.416946778	0.7416444440194403	0.665585920442097
	annotation_met	0.699657869012707	0.25	0.7905669599217986	0.7891472326956198
	annotation_rxn	0.716940815997419	0.285377358	0.8076822286727947	0.7579321883567167
	annotation_gene	0.424010025062656	0	0.4240100250626566	0.456516290726817
	annotation_sbo	0.272793244491357	0	0.5454545454545454	0.36363636363636365
iECABU_c1320	consistency	0.971339093198364	0.971339093	0.9714612977688157	0.9714612977688157

	total_score	0.614136380495777	0.417424552	0.7387096726397014	0.662415993048611
	annotation_met	0.699255687669693	0.25	0.7901647785787848	0.7887487126673532
	annotation_rxn	0.716689043492412	0.285701208	0.807243988770902	0.757445380202612
	annotation_gene	0.366666666666666	0	0.3666666666666667	0.395
	annotation_sbo	0.272793848407176	0	0.5454545454545454	0.36363636363636365
iECBD_1354	consistency	0.971854729096077	0.971809984	0.9718547290960773	0.9718099841790077
	total_score	0.614371325393356	0.417591316	0.7389104249445864	0.6625244342644426
	annotation_met	0.699445789865871	0.25	0.7903548807749627	0.7889460692995529
	annotation_rxn	0.716965874171114	0.285480349	0.8076985282225457	0.7580058224163028
	annotation_gene	0.366666666666666	0	0.3666666666666667	0.3935007385524372
	annotation_sbo	0.272793436548895	0	0.5454545454545454	0.36363636363636365
iECB_1328	consistency	0.972031775976299	0.971898976	0.9720757275588741	0.9718989760332534
	total_score	0.614441480872781	0.417625924	0.7389984214736519	0.6627680867898662
	annotation_met	0.699443175993663	0.25	0.7903522669027538	0.7889427333302269
	annotation_rxn	0.716986090894387	0.285480349	0.8077288533074559	0.7580260391395762
	annotation_gene	0.366666666666666	0	0.3666666666666667	0.3972410333584149
	annotation_sbo	0.272793436548895	0	0.5454545454545454	0.36363636363636365
iECDH10B_1368	consistency	0.971272606755826	0.971272607	0.9715158713669074	0.9715158713669074
	total_score	0.617788826515571	0.417388087	0.7424154148603672	0.6660042064900061
	annotation_met	0.699432693654573	0.25	0.7903417845636643	0.7889293551851333
	annotation_rxn	0.716792284625982	0.285557987	0.8074296944647055	0.7576789042872194
	annotation_gene	0.432504395880432	0	0.43250439588043205	0.45865360462195426
	annotation_sbo	0.272793581327498	0	0.5454545454545454	0.36363636363636365
iECDH1ME8569_1439	consistency	0.971394335636230	0.971439677	0.9716362613650313	0.9716816023706125
	total_score	0.614196270197579	0.41744063	0.7388273809753811	0.6625886779296882
	annotation_met	0.699487179487179	0.25	0.7903962703962704	0.788962703962704
	annotation_rxn	0.716979229683403	0.2853902	0.8076830006049607	0.7579048195200645
	annotation_gene	0.366666666666666	0	0.36666666666666664	0.39566828816307625
	annotation_sbo	0.272793268437551	0	0.5454545454545454	0.36363636363636365
iEcDH1_1363	consistency	0.971494431104747	0.97153961	0.9717368700404398	0.9717820491138581
	total_score	0.614240965178640	0.417484259	0.7388739675256865	0.6627405721167291
	annotation_met	0.699472923177387	0.25	0.7903820140864779	0.7889477121134381
	annotation_rxn	0.717070707070707	0.285454545	0.8077979797979797	0.758010101010101
	annotation_gene	0.366666666666666	0	0.36666666666666664	0.39757887013939847
	annotation_sbo	0.272793388429752	0	0.5454545454545454	0.36363636363636365

iECD_1391	consistency	0.972180145036885	0.972180145	0.9721801450368857	0.9721801450368857
	total_score	0.614492082286453	0.417741979	0.7390287593384328	0.6628255722701956
	annotation_met	0.699410471155195	0.25	0.7903195620642867	0.7889159219576101
	annotation_rxn	0.716922047914386	0.28557096	0.8076229275609064	0.7579654627264989
	annotation_gene	0.366666666666666	0	0.3666666666666664	0.39642410602650663
	annotation_sbo	0.272793605518888	0	0.5454545454545454	0.36363636363636365
iEcE24377_1341	consistency	0.970234739160210	0.970190155	0.9705964028130133	0.9705518182719903
	total_score	0.613711963003294	0.416947114	0.7383911144189628	0.6622491730442361
	annotation_met	0.699243960907246	0.25	0.7901530518163378	0.7887354785174258
	annotation_rxn	0.716773233602766	0.285287731	0.8074958780713395	0.757871878393051
	annotation_gene	0.366666666666666	0	0.3666666666666667	0.3978125776783495
	annotation_sbo	0.272793077353337	0	0.5454545454545454	0.36363636363636365
iECED1_1282	consistency	0.971971548127976	0.971971548	0.9723421243740394	0.9723421243740394
	total_score	0.617625685089301	0.417694939	0.7423048200795451	0.6660624448455152
	annotation_met	0.699915170366181	0.25	0.7908242612752722	0.7894104340449597
	annotation_rxn	0.716945881580027	0.286031042	0.8076291368974295	0.7577810626591115
	annotation_gene	0.423820693249934	0	0.4238206932499349	0.45314047432890275
	annotation_sbo	0.272794463481824	0	0.5454545454545454	0.36363636363636365
iECH74115_1262	consistency	0.974520123450381	0.974520123	0.9747684626817719	0.9747684626817719
	total_score	0.615430008303687	0.41869794	0.7400532169931829	0.663858194526509
	annotation_met	0.699853066641387	0.25	0.7907621575504787	0.7893283723575695
	annotation_rxn	0.716850202095191	0.286191537	0.8074218427781902	0.7576198135774973
	annotation_gene	0.366666666666666	0	0.3666666666666667	0.39680401479133653
	annotation_sbo	0.272794762772491	0	0.5454545454545454	0.36363636363636365
iEcHS_1320	consistency	0.971540344640580	0.971540345	0.9714798119529291	0.9714798119529291
	total_score	0.614216174551332	0.417481683	0.7387352715598915	0.6626321144420659
	annotation_met	0.699103876256194	0.25	0.790012967165285	0.788669939332191
	annotation_rxn	0.716864430722040	0.28541591	0.807644186140372	0.7580518222545103
	annotation_gene	0.366666666666666	0	0.3666666666666664	0.3980570275044159
	annotation_sbo	0.272793316382128	0	0.5454545454545454	0.36363636363636365
iECIAI1_1343	consistency	0.394941513190642	0.394941513	0.537798656047785	0.537798656047785
	total_score	0.393133129491293	0.193237419	0.5732230921410312	0.49714517998464725
	annotation_met	0.699140798226164	0.25	0.790049889135255	0.7886640798226164
	annotation_rxn	0.716425557564798	0.285262206	0.8070926260799678	0.757655213984328

	annotation_gene	0.423901712583767	0	0.42390171258376763	0.45559692231322907
	annotation_sbo	0.272793029755055	0	0.5454545454545454	0.36363636363636365
iECIAI39_1322	consistency	0.969616332418003	0.969518575	0.9698005057865341	0.9697027486889616
	total_score	0.616700497093487	0.416726291	0.778345865885789	0.7021422547158033
	annotation_met	0.699518223711772	0.25	0.790427314620863	0.7889959502862728
	annotation_rxn	0.717219976315897	0.285832415	0.8079239658622239	0.7579933847850056
	annotation_gene	0.423820338127681	0	0.42382033812768105	0.4546555639666919
	annotation_sbo	0.272794093080752	0	0.6363636363636364	0.45454545454545453
iECNA114_1301	consistency	0.972057629792063	0.972102809	0.9722419615892984	0.9722871406627167
	total_score	0.614509879596329	0.417734201	0.7391158174077923	0.6630477511243336
	annotation_met	0.700110864745011	0.25	0.791019955654102	0.7895928669151295
	annotation_rxn	0.717102035810645	0.285871965	0.8077732810072766	0.7578897882429891
	annotation_gene	0.366666666666666	0	0.3666666666666667	0.3988726620548297
	annotation_sbo	0.272794166833901	0	0.5454545454545454	0.36363636363636365
iECO103_1326	consistency	0.969509102090870	0.969509102	0.9695695068024375	0.9695695068024375
	total_score	0.613470712000417	0.416686999	0.7380366350756338	0.661881927768447
	annotation_met	0.699705172253691	0.25	0.790614263162782	0.7892213761723466
	annotation_rxn	0.716864072194021	0.285351704	0.8076403996454758	0.7580170816211426
	annotation_gene	0.366666666666666	0	0.36666666666666664	0.39723687515699574
	annotation_sbo	0.272793196651064	0	0.5454545454545454	0.36363636363636365
iECO111_1330	consistency	0.969908847360712	0.969908847	0.9698484936881916	0.9698484936881916
	total_score	0.613594180496740	0.416840558	0.7381067978276538	0.6618060041342536
	annotation_met	0.699440809318297	0.25	0.7903499002273887	0.788957724256346
	annotation_rxn	0.716696859903381	0.285326087	0.8073872785829308	0.7578603059581321
	annotation_gene	0.366666666666666	0	0.3666666666666667	0.3944779116465864
	annotation_sbo	0.272793148880105	0	0.5454545454545454	0.36363636363636365
iECO26_1355	consistency	0.969352741826748	0.969352742	0.9693065663239733	0.9693527418267484
	total_score	0.613367334598607	0.416605469	0.7378891395883292	0.6616552422610213
	annotation_met	0.699340735600277	0.25	0.7902498265093685	0.7888503354152209
	annotation_rxn	0.716656674660271	0.285071942	0.8073940847322142	0.7578437250199841
	annotation_gene	0.366666666666666	0	0.3666666666666667	0.39539975399754
	annotation_sbo	0.272792674950948	0	0.5454545454545454	0.36363636363636365
iECOK1_1307	consistency	0.969629934722328	0.969582135	0.9697522439199796	0.9697044446945949
	total_score	0.613478351278713	0.416743228	0.7380550721924374	0.6616576636732134

	annotation_met	0.699288089550840	0.25	0.7901971804599316	0.7887686759402369
	annotation_rxn	0.716746060828142	0.285727373	0.8073470135580799	0.7575119091242214
	annotation_gene	0.366666666666666	0	0.36666666666666664	0.39353271983640087
	annotation_sbo	0.272793897198441	0	0.5454545454545454	0.36363636363636365
iEcolC_1368	consistency	0.970815549386051	0.970815549	0.9710561508898105	0.9710561508898105
	total_score	0.613925945941260	0.417185602	0.7385622808392156	0.6623395718156244
	annotation_met	0.699074287824922	0.25	0.7899833787340136	0.7885867306893208
	annotation_rxn	0.716783076429030	0.285223988	0.8075626204238922	0.7579680475272961
	annotation_gene	0.366666666666666	0	0.3666666666666667	0.3959795321637427
	annotation_sbo	0.272792958486600	0	0.5454545454545454	0.36363636363636365
iECP_1309	consistency	0.969718260524679	0.96976606	0.9699618362158253	0.9700096354412098
	total_score	0.613502759097125	0.416805092	0.7379850203794399	0.6617562519819468
	annotation_met	0.699358343871481	0.25	0.7902674347805723	0.7888506393143178
	annotation_rxn	0.716542939434505	0.285596933	0.8071376414749909	0.757342501318405
	annotation_gene	0.366666666666666	0	0.3661581489956776	0.3932875667429443
	annotation_sbo	0.272793653954661	0	0.5451771721794606	0.36363636363636365
iECS88_1305	consistency	0.969894526815747	0.969894527	0.9700168360133992	0.9700168360133992
	total_score	0.616738073738815	0.416864714	0.7413117787299768	0.6650315822758314
	annotation_met	0.699337608838123	0.25	0.7902466997472146	0.7888306338357831
	annotation_rxn	0.716593379748381	0.285727373	0.8071536175237164	0.7573694067831115
	annotation_gene	0.423627075351213	0	0.4236270753512133	0.452183908045977
	annotation_sbo	0.272793897198441	0	0.5454545454545454	0.36363636363636365
iECSE_1348	consistency	0.969584685738307	0.969584686	0.9695245353623679	0.9695245353623679
	total_score	0.613498285860275	0.416706932	0.7380176616939025	0.661984597980998
	annotation_met	0.699702698936219	0.25	0.7906117898453106	0.7892181911088401
	annotation_rxn	0.716843288375080	0.285223988	0.8076228323699421	0.7579780828516378
	annotation_gene	0.366666666666666	0	0.3666666666666667	0.39945598417408507
	annotation_sbo	0.272792958486600	0	0.5454545454545454	0.36363636363636365
iECSF_1327	consistency	0.971278377251283	0.971278377	0.9714608257095941	0.9714608257095941
	total_score	0.614108533664506	0.417390331	0.7387114712832253	0.6626368043493813
	annotation_met	0.699117002935557	0.25	0.7900260938446484	0.788628209309911
	annotation_rxn	0.716772023664802	0.285557987	0.8074094335035255	0.7576282518842694
	annotation_gene	0.366666666666666	0	0.3666666666666667	0.39889475006279834
	annotation_sbo	0.272793581327498	0	0.5454545454545454	0.36363636363636365

iEcSMS35_1347	consistency	0.971329288196366	0.971239091	0.971632852035842	0.971542654955221
	total_score	0.614171805877015	0.417371216	0.7388293375970889	0.6626440560781213
	annotation_met	0.699514404445067	0.25	0.7904234953541579	0.7890227389456973
	annotation_rxn	0.716962045803997	0.285506191	0.8077000890183701	0.7579104960750991
	annotation_gene	0.366666666666666	0	0.36666666666666664	0.39755011135857465
	annotation_sbo	0.272793484738131	0	0.5454545454545454	0.36363636363636365
iECSP_1301	consistency	0.973608688268380	0.973608688	0.9739783057143238	0.9739783057143238
	total_score	0.615076223967511	0.418325699	0.7397485311942472	0.6633863855196657
	annotation_met	0.699869791666666	0.25	0.7904234953541579	0.7893465909090909
	annotation_rxn	0.716844886922320	0.285951327	0.8077000890183701	0.7576409373975745
	annotation_gene	0.366666666666666	0	0.36666666666666664	0.3937900949448294
	annotation_sbo	0.272794314829713	0	0.5454545454545454	0.36363636363636365
iECs_1301	consistency	0.973544740025187	0.973458164	0.9737903045165616	0.9737037286614505
	total_score	0.615031890599035	0.41825933	0.7396544532167011	0.6632969606223609
	annotation_met	0.699735262137758	0.25	0.7906443530468491	0.789214295844561
	annotation_rxn	0.716717728758170	0.285845588	0.8072916666666666	0.7575776143790849
	annotation_gene	0.366666666666666	0	0.3666666666666667	0.39436331027414817
	annotation_sbo	0.272794117647058	0	0.5454545454545454	0.36363636363636365
iECUMN_1333	consistency	0.972078303717087	0.971989929	0.972078303717087	0.9719899287997108
	total_score	0.617666776715075	0.417668968	0.7422073978493706	0.6660780956763527
	annotation_met	0.699894291754756	0.25	0.7908033826638478	0.789405684754522
	annotation_rxn	0.716869424168694	0.285583942	0.8076236820762368	0.757978507704785
	annotation_gene	0.423948948948948	0	0.42394894894894897	0.45563063063063064
	annotation_sbo	0.272793629727936	0	0.5454545454545454	0.36363636363636365
iECW_1372	consistency	0.970178404209036	0.970134138	0.9702382020921917	0.9701939357273274
	total_score	0.613686342339432	0.416907478	0.7382541613878822	0.6621212447067661
	annotation_met	0.699189052204764	0.25	0.7900981431138552	0.7887043265907939
	annotation_rxn	0.716780493649652	0.285046729	0.8075824746385494	0.758017812924355
	annotation_gene	0.366666666666666	0	0.36666666666666664	0.3978620019436347
	annotation_sbo	0.272792627932814	0	0.5454545454545454	0.36363636363636365
iEK1008	consistency	0.515652440013177		0.6585095828703205	
	total_score	0.438522501210511		0.6205055919956185	
	annotation_met	0.663121697941337		0.7795363454181089	
	annotation_rxn	0.724646547036432		0.8154114555011782	
	annotation_gene	0.432936507936507		0.43293650793650795	

	annotation_sbo	0.272801423698650		0.5454545454545454	
iEKO11_1354	consistency	0.970365978626647	0.970365979	0.9704258767997535	0.9704258767997535
	total_score	0.613753648504528	0.417001376	0.7383207834450225	0.6621763349517044
	annotation_met	0.699140236031716	0.25	0.7900493269408077	0.7886663286004056
	annotation_rxn	0.716752659787217	0.285097192	0.8075453963682906	0.7580093592512599
	annotation_gene	0.366666666666666	0	0.3666666666666664	0.3972919743968489
	annotation_sbo	0.272792722036782	0	0.5454545454545454	0.36363636363636365
iETEC_1333	consistency	0.970265001027745	0.970265001	0.9705068243757895	0.9705068243757895
	total_score	0.617405729138420	0.41698286	0.7418936516605413	0.665839409519072
	annotation_met	0.699089519043647	0.25	0.7899986099527384	0.7885853952367714
	annotation_rxn	0.716840025802290	0.285377358	0.807520964360587	0.7578011611030478
	annotation_gene	0.433058264566141	0	0.43250936329588013	0.46304619225967536
	annotation_sbo	0.272793244491357	0	0.5451821586653047	0.36363636363636365
iG2583_1286	consistency	0.973749818491884	0.973749818	0.9741207154443482	0.9741207154443482
	total_score	0.615147617702617	0.418388462	0.7398226941465935	0.6636736446975768
	annotation_met	0.699973944762897	0.25	0.7908830356719883	0.789449997631342
	annotation_rxn	0.716962524654832	0.286057692	0.8075895792241946	0.7577251808021038
	annotation_gene	0.366666666666666	0	0.3666666666666667	0.3977136918680177
	annotation_sbo	0.272794513179128	0	0.5454545454545454	0.36363636363636365
iHN637	consistency	0.854306983339241	0.854306983	0.9971641261963843	0.9971641261963843
	total_score	0.570414136548447	0.371555806	0.7511357189685439	0.6745614927716252
	annotation_met	0.705978119301901	0.25	0.7968872102109924	0.7945754102630893
	annotation_rxn	0.733864118895966	0.28089172	0.8333333333333334	0.7812101910828027
	annotation_gene	0.366666666666666	0	0.3666666666666664	0.3942438513867086
	annotation_sbo	0.272843080486392	0	0.5454545454545454	0.36363636363636365
iIS312	consistency	0.683737773854052		0.7589416397555933	
	total_score	0.497491804466095		0.6161635328309482	
	annotation_met	0.662803780378037		0.788966396639664	
	annotation_rxn	0.712748876043673		0.7947976878612717	
	annotation_gene	0.333333333333333		0.3333333333333333	
	annotation_sbo	0.272902434752145		0.45454545454545453	
iIS312_Amastigote	consistency	0.707268590863130		0.7824724567646711	
	total_score	0.506642677747403		0.6253144061122563	
	annotation_met	0.662803780378037		0.788966396639664	

	annotation_rxn	0.712748876043673		0.7947976878612717	
	annotation_gene	0.333333333333333		0.3333333333333333	
	annotation_sbo	0.272902434752145		0.45454545454545453	
iIS312_Epimastigote	consistency	0.683004284042893		0.7582081499444335	
	total_score	0.497206558428422		0.6158782867932749	
	annotation_met	0.662803780378037		0.788966396639664	
	annotation_rxn	0.712748876043673		0.7947976878612717	
	annotation_gene	0.333333333333333		0.3333333333333333	
	annotation_sbo	0.272902434752145		0.45454545454545453	
iIS312_Trypomastigote	consistency	0.697942686588377		0.7731465524899177	
	total_score	0.503080460614840		0.6216773271755445	
	annotation_met	0.662803780378037		0.788966396639664	
	annotation_rxn	0.712660256410256		0.7946581196581196	
	annotation_gene	0.333333333333333		0.3333333333333333	
	annotation_sbo	0.273076923076923		0.45454545454545453	
iIT341	consistency	0.846570364672815	0.848271419	0.989427507529958	0.9911285615163496
	total_score	0.572232692778871	0.369460813	0.7897754826241083	0.7137592626165343
	annotation_met	0.713964386129334	0.25	0.8048734770384255	0.8033739456419868
	annotation_rxn	0.741075010028078	0.284296029	0.8378961091054953	0.7824909747292419
	annotation_gene	0.432940019665683	0	0.4329400196656834	0.46194690265486726
	annotation_sbo	0.272891368559238	0	0.6363636363636364	0.45454545454545453
iJB785	consistency	0.968717128784693			
	total_score	0.611402738911632			
	annotation_met	0.666074810606060			
	annotation_rxn	0.721796885224447			
	annotation_gene	0.366666666666664			
	annotation_sbo	0.273690973376164			
iJN1463	consistency	0.973337304664587		0.9732822405670987	
	total_score	0.611450005714282		0.7765372129999512	
	annotation_met	0.615705358273867		0.7643562893214542	
	annotation_rxn	0.703849219906616		0.7845063204646395	
	annotation_gene	0.432877336981304		0.43287733698130415	
	annotation_sbo	0.272789390315867		0.6363636363636364	
iJN678	consistency	0.996088905936411	0.996090754	0.9960889059364118	0.996090754412228

	total_score	0.625128950289469	0.425521496	0.7869910842048412	0.7110082735901526
	annotation_met	0.701543739279588	0.25	0.7924528301886792	0.7897084048027444
	annotation_rxn	0.731492210634736	0.265063731	0.8314664606669242	0.7811896485129394
	annotation_gene	0.366666666666666	0	0.36559829059829063	0.39764957264957274
	annotation_sbo	0.273043295059517	0	0.6357808857808859	0.45454545454545453
iJN746	consistency	0.829958884032218	0.829958884	0.9728160268893612	0.9728160268893612
	total_score	0.564800817255383	0.3615558	0.7824870264554025	0.7057126831706482
	annotation_met	0.708830309712338	0.25	0.7997394006214292	0.7980855968728074
	annotation_rxn	0.733791903858317	0.273719165	0.8321210204511913	0.7789900906599199
	annotation_gene	0.432573726541554	0	0.43257372654155496	0.4570151921358356
	annotation_sbo	0.272813524236674	0	0.6363636363636364	0.45454545454545453
iJO1366	consistency	0.981837896448902	0.981837896	0.9818033677349266	0.9818378964489022
	total_score	0.633295335057560	0.421228782	0.7578726186864839	0.6818102478554292
	annotation_met	0.700818433643918	0.25	0.7917275245530093	0.7903173004281038
	annotation_rxn	0.718705639437346	0.281939605	0.8101582999956983	0.7597647008216114
	annotation_gene	0.633235796147281	0	0.6332357961472811	0.6662765179224579
	annotation_sbo	0.272797663041565	0	0.5454545454545454	0.36363636363636365
iJR904	consistency	0.976186028514053	0.976143718	0.9761860285140536	0.9761011763265641
	total_score	0.633761831467501	0.419111877	0.7216997811625567	0.6449437613388722
	annotation_met	0.715326723211085	0.25	0.8062358141201768	0.8046828335921634
	annotation_rxn	0.741343669250646	0.283255814	0.8380620155038759	0.7825322997416021
	annotation_gene	0.631563421828908	0	0.6315634218289086	0.6599926253687315
	annotation_sbo	0.272811839323467	0	0.45454545454545453	0.2727272727272727
iLB1027_lipid	consistency	0.996125892813369	0.996190703	0.9956082882909504	0.9960286007751008
	total_score	0.611863793032080	0.424614923	0.7788003795865424	0.6965034625777046
	annotation_met	0.556713544282605	0.25	0.7460865561694291	0.7450192533065462
	annotation_rxn	0.680985687213245	0.252300269	0.7487594753640534	0.71645596449232
	annotation_gene	0.389354105809802	0	0.389354105809802	0.28289516390782216
	annotation_sbo	0.272870083238126	0	0.6363636363636364	0.45454545454545453
iLF82_1304	consistency	0.970114380213239	0.97011438	0.9702368466880419	0.9702368466880419
	total_score	0.617372046370895	0.416953125	0.7419501458396629	0.6657276448531456
	annotation_met	0.699432404540763	0.25	0.7903414954498545	0.7889225068017637
	annotation_rxn	0.716872503464579	0.285766691	0.8074916442487976	0.7576526453085515
	annotation_gene	0.433000512032770	0	0.43300051203277007	0.4626728110599078
	annotation_sbo	0.272793970519575	0	0.5454545454545454	0.36363636363636365

iLJ478	consistency	0.847633090880553	0.847633091	0.9904902337376967	0.9904902337376967
	total_score	0.571208873406484	0.368944339	0.7888135156021284	0.7127576583800695
	annotation_met	0.703708133971291	0.25	0.7946172248803828	0.7935007974481658
	annotation_rxn	0.731978527607362	0.280674847	0.8294989775051125	0.7811434901158828
	annotation_gene	0.433056708160442	0	0.4330567081604426	0.46334716459197783
	annotation_sbo	0.272866703848298	0	0.6363636363636364	0.45454545454545453
iML1515	consistency	0.984066783055405	0.984036317	0.98403631661419	0.98403631661419
	total_score	0.633025729725349	0.422018985	0.7581175294746013	0.6821437996626633
	annotation_met	0.688066062866276	0.25	0.7878142102968955	0.7863733230009202
	annotation_rxn	0.716127908882333	0.281065634	0.805647738446411	0.7566576532284497
	annotation_gene	0.633245382585752	0	0.633245382585752	0.6662928759894459
	annotation_sbo	0.272794314829713	0	0.5454545454545454	0.36363636363636365
iMM1415	consistency	0.414134322292217	0.414134322	0.5493415148391452	0.5493415148391452
	total_score	0.402522705559525	0.200310899	0.6164360392130475	0.5408110717079823
	annotation_met	0.667698607698607	0.25	0.7586895986895987	0.7568959868959869
	annotation_rxn	0.693236714975845	0.279991948	0.7803721595992128	0.7427014373471701
	annotation_gene	0.531709090909090	0	0.5317090909090909	0.5564121212121212
	annotation_sbo	0.272751671302395	0	0.6363636363636364	0.45454545454545453
iMM904	consistency	0.958299097817511	0.958213248	0.9582990978175115	0.9582132483645734
	total_score	0.613712541163937	0.411601354	0.701463272365666	0.6233982608355999
	annotation_met	0.705379652973454	0.25	0.7962887438825449	0.794620347026546
	annotation_rxn	0.723490453040231	0.275998732	0.8175332910589728	0.7669625871908687
	annotation_gene	0.433149171270718	0	0.4331491712707183	0.43156537753222834
	annotation_sbo	0.272784919582636	0	0.45454545454545453	0.2727272727272727
iND750	consistency	0.830161895559399	0.830098746	0.9722115357701048	0.9720855663964089
	total_score	0.564300836810498	0.361550015	0.7074366950904397	0.6293004272356824
	annotation_met	0.706391106532749	0.25	0.7973001974418404	0.7954330843849258
	annotation_rxn	0.728058627347726	0.272906793	0.8241179568193786	0.7729067930489731
	annotation_gene	0.433155555555555	0	0.43315555555555557	0.43168888888888884
	annotation_sbo	0.272799080855952	0	0.45454545454545453	0.2727272727272727
iNF517	consistency	0.974814423450987	0.974814423	0.9745936241575456	0.9745936241575456
	total_score	0.611779804755327	0.418710374	0.7014300359539364	0.6049586713599665
	annotation_met	0.664055944055944	0.25	0.7888811188811189	0.7870629370629371
	annotation_rxn	0.725869437076333	0.284814324	0.8138078396699087	0.763262599469496

	annotation_gene	0.333333333333333	0	0.3333333333333333	0.0
	annotation_sbo	0.272968410899445	0	0.45454545454545453	0.2727272727272727
iNJ661	consistency	0.737549636758859	0.737446573	0.8804067796160027	0.8803037155204416
	total_score	0.528163900552512	0.325393631	0.7087210529253828	0.6321810157762339
	annotation_met	0.699862258953168	0.25	0.7907713498622589	0.789366391184573
	annotation_rxn	0.732953929539295	0.271219512	0.8300542005420054	0.7762059620596206
	annotation_gene	0.433030761472516	0	0.4330307614725164	0.46303580433686337
	annotation_sbo	0.272815964523281	0	0.5454545454545454	0.36363636363636365
iNRG857_1313	consistency	0.970128969515745	0.97012897	0.9702509653678868	0.9702509653678868
	total_score	0.613685097083047	0.41695008	0.73826207933327	0.6619644184542431
	annotation_met	0.699387077153417	0.25	0.7902961680625088	0.7888808309549431
	annotation_rxn	0.716819012797074	0.285648995	0.8074243347552306	0.7575969937030267
	annotation_gene	0.366666666666666	0	0.3666666666666667	0.3949656750572083
	annotation_sbo	0.272793751038723	0	0.5454545454545454	0.36363636363636365
iPC815	consistency	0.993570927830014	0.993570928	0.9935709278300143	0.9935709278300143
	total_score	0.630616084146259	0.426079328	0.7181531161277364	0.6419481466846112
	annotation_met	0.702597820993439	0.25	0.7935069119025304	0.7918082239925023
	annotation_rxn	0.719176723893705	0.285823559	0.8105275086407162	0.7587965323814381
	annotation_gene	0.499713701431492	0	0.4997137014314928	0.5325971370143149
	annotation_sbo	0.272819989801121	0	0.45454545454545453	0.2727272727272727
iRC1080	consistency	0.764601165248125	0.764693971	0.9283827262707615	0.928289920085079
	total_score	0.531839536366942	0.335018124	0.7426543794422039	0.6614762390401471
	annotation_met	0.699629649365874	0.25	0.7905387402749653	0.7886070553127997
	annotation_rxn	0.715363355139712	0.258101324	0.8112860692732897	0.7678000912825194
	annotation_gene	0.333333333333333	0	0.3333333333333333	0.0
	annotation_sbo	0.272851748890087	0	0.6	0.45454545454545453
iSbBS512_1146	consistency	0.973916510352758	0.973874571	0.9740464986538111	0.9740045597816236
	total_score	0.615172490642322	0.418553464	0.7397278059746568	0.6628964525034564
	annotation_met	0.699583531651594	0.25	0.7904926225606853	0.7890409328891004
	annotation_rxn	0.716797461297654	0.287630259	0.8070886401646726	0.757086495990394
	annotation_gene	0.366666666666666	0	0.3666666666666667	0.38593432141819245
	annotation_sbo	0.272797445703659	0	0.5454545454545454	0.36363636363636365
iSBO_1134	consistency	0.979580168921245	0.979580169	0.979580168921245	0.979580168921245
	total_score	0.617340932976976	0.420772307	0.77846832854419	0.7022481689909477

	annotation_met	0.699423480083857	0.25	0.7903325709929483	0.788891271202592
	annotation_rxn	0.716497276898666	0.287630259	0.8066812470517604	0.7568720785625456
	annotation_gene	0.366666666666666	0	0.3652033947907521	0.38905472636815924
	annotation_sbo	0.272797445703659	0	0.635565488067683	0.45454545454545453
iSDY_1059	consistency	0.979154179154179	0.979154179	0.9792873792873793	0.9792873792873793
	total_score	0.617120441581975	0.420663732	0.7785089545543232	0.7018329483794914
	annotation_met	0.699284957627118	0.25	0.7901940485362096	0.7887856317411402
	annotation_rxn	0.715887707321342	0.288400945	0.8057743643604218	0.75610476565577
	annotation_gene	0.366666666666666	0	0.366038328620798	0.38479421928997803
	annotation_sbo	0.272798882881592	0	0.6360209065204352	0.45454545454545453
iSFV_1184	consistency	0.974301986405306	0.974301986	0.9743019864053065	0.9743019864053065
	total_score	0.615341145354796	0.418687776	0.739696755812698	0.6633131335536285
	annotation_met	0.699874330156020	0.25	0.7907834210651112	0.7893488879404372
	annotation_rxn	0.716764169740133	0.287199542	0.8071452795794649	0.7572491415490271
	annotation_gene	0.366666666666666	0	0.3661045531197302	0.3907251264755481
	annotation_sbo	0.272796642502861	0	0.5451479380653074	0.36363636363636365
iSFxv_1172	consistency	0.975291953901219	0.975291954	0.9752919539012191	0.9752919539012191
	total_score	0.615684374738768	0.419055006	0.777230762719324	0.7009965910985861
	annotation_met	0.699888615034600	0.25	0.7907977059436914	0.789363920750782
	annotation_rxn	0.716188610900513	0.286959818	0.8065348327857804	0.756865470474265
	annotation_gene	0.366666666666666	0	0.3666666666666667	0.3959224408326205
	annotation_sbo	0.272796195464883	0	0.6363636363636364	0.45454545454545453
iSF_1195	consistency	0.973983561354215	0.973940529	0.9739835613542152	0.9739405291485213
	total_score	0.615207455472977	0.418537779	0.7767545558244712	0.7001021360890612
	annotation_met	0.699850618864703	0.25	0.7907597097737943	0.7893251766491203
	annotation_rxn	0.716656104773975	0.287072243	0.807013096746937	0.7571504013519222
	annotation_gene	0.366666666666666	0	0.36666666666666664	0.38895397489539746
	annotation_sbo	0.272796405115796	0	0.6363636363636364	0.45454545454545453
iSSON_1240	consistency	0.972956875946315	0.972912219	0.9729568759463153	0.9729122188304465
	total_score	0.614737167465549	0.418073639	0.7762956772932341	0.699898220731678
	annotation_met	0.699286250939143	0.25	0.7901953418482344	0.7888101051840721
	annotation_rxn	0.716270578041836	0.286204976	0.8067727028922721	0.7571275322853488
	annotation_gene	0.366666666666666	0	0.3666666666666667	0.39319892473118284
	annotation_sbo	0.272794787833777	0	0.6363636363636364	0.45454545454545453

iSynCJ816	consistency	0.757225971033943		0.7571753174621632	
	total_score	0.528327145928181		0.6918936964981169	
	annotation_met	0.660609326018808		0.7794376959247649	
	annotation_rxn	0.719934014474244		0.8109567901234568	
	annotation_gene	0.366666666666666		0.3666666666666664	
	annotation_sbo	0.272988505747126		0.6363636363636364	
iS_1188	consistency	0.975036393140438	0.975036393	0.9750363931404389	0.9750363931404389
	total_score	0.615615423276580	0.418975482	0.7771614479170859	0.70062348969375
	annotation_met	0.699831385959912	0.25	0.7907404768690035	0.7893036952598081
	annotation_rxn	0.716653939162530	0.28722795	0.806998006024352	0.7571167960629587
	annotation_gene	0.366666666666666	0	0.3666666666666667	0.3907407407407408
	annotation_sbo	0.272796695477107	0	0.6363636363636364	0.45454545454545453
iUMN146_1321	consistency	0.969455557945658	0.969407759	0.9695775537977998	0.9695297545724154
	total_score	0.613407169568717	0.416669609	0.73798339949237	0.6616069026256818
	annotation_met	0.699302499765939	0.25	0.7902115906750304	0.7887838217395375
	annotation_rxn	0.716686979484054	0.285648995	0.8072821450335161	0.7574649603900061
	annotation_gene	0.366666666666666	0	0.3666666666666667	0.393884255749305
	annotation_sbo	0.272793751038723	0	0.5454545454545454	0.36363636363636365
iUMNK88_1353	consistency	0.9692	0.9692	0.9695	0.9694472289120215
	total_score	0.6133	0.4165	0.7379	0.6618059893652245
	annotation_met	0.6991	0.25	0.79	0.7885867306893208
	annotation_rxn	0.7167	0.2851	0.8074	0.7578221902132597
	annotation_gene	0.3667	0	0.3667	0.39783197831978323
	annotation_sbo	0.2728	0	0.5455	0.36363636363636365
iUTI89_1310	consistency	0.971207927658125	0.971162587	0.9713304466485686	0.9712851056429875
	total_score	0.614091371561949	0.417361733	0.73866439392102	0.6624878530647225
	annotation_met	0.699297094657919	0.25	0.7902061855670103	0.7887769447047798
	annotation_rxn	0.716727828746177	0.285779817	0.8072782874617738	0.7574515800203874
	annotation_gene	0.366666666666666	0	0.3666666666666667	0.39748091603053437
	annotation_sbo	0.272793994995829	0	0.5454545454545454	0.36363636363636365
iWFL_1372	consistency	0.970089712818502	0.970134138	0.970149510701657	0.9701939357273274
	total_score	0.613651851243113	0.416907478	0.7382196702915633	0.6621212447067661
	annotation_met	0.699189052204764	0.25	0.7900981431138552	0.7887043265907939
	annotation_rxn	0.716780493649652	0.285046729	0.8075824746385494	0.758017812924355
	annotation_gene	0.366666666666666	0	0.3666666666666664	0.3978620019436347

	annotation_sbo	0.272792627932814	0	0.5454545454545454	0.36363636363636365
iY75_1357	consistency	0.971263948700615	0.971263949	0.9715054654256488	0.9715054654256488
	total_score	0.614150508937959	0.41736849	0.738782945455663	0.6626632927805678
	annotation_met	0.699506586603360	0.25	0.7904156775124517	0.7889843131778616
	annotation_rxn	0.717027103217752	0.285338891	0.8077503926543433	0.7579839716483427
	annotation_gene	0.366666666666666	0	0.3666666666666667	0.3981099656357389
	annotation_sbo	0.272793172756927	0	0.5454545454545454	0.36363636363636365
iYL1228	consistency	0.700245700245700	0.7002457	0.843102843102843	0.843102843102843
	total_score	0.509362462827685	0.311720792	0.6896163889309087	0.6134690068729859
	annotation_met	0.703421427788134	0.25	0.7943305186972256	0.7926993091347735
	annotation_rxn	0.721460850771195	0.28194076	0.814200805580116	0.7630661165143924
	annotation_gene	0.366666666666666	0	0.36666666666666664	0.3997016544616219
	annotation_sbo	0.272767462422634	0	0.5454545454545454	0.36363636363636365
iYO844	consistency	0.982279469164715	0.9823	0.9822794691647151	0.9823018035552096
	total_score	0.619610576817465	0.4224	0.7072219498066161	0.6308505556547944
	annotation_met	0.705486685032139	0.25	0.7963957759412305	0.7939164370982552
	annotation_rxn	0.726888888888888	0.2958	0.8191333333333334	0.7658666666666667
	annotation_gene	0.366666666666666	0	0.3666666666666667	0.3994865718799368
	annotation_sbo	0.272800000000000	0	0.45454545454545453	0.2727272727272727
iYS1720	consistency	0.907726463194649		0.9080253275102503	
	total_score	0.590491466918338		0.7183247268158668	
	annotation_met	0.624216300940438		0.7653194506642782	
	annotation_rxn	0.705863370072485		0.7895359613411446	
	annotation_gene	0.500527240773286		0.5005272407732865	
	annotation_sbo	0.272835594551412		0.5454545454545454	
iYS854	consistency	0.680730451060771			
	total_score	0.492919661730758			
	annotation_met	0.619526727953694			
	annotation_rxn	0.706586483390607			
	annotation_gene	0.333333333333333			
	annotation_sbo	0.273539518900343			
iZ_1308	consistency	0.973631775351685	0.973459245	0.9738772343600307	0.9737047043794682
	total_score	0.615065121313955	0.418258774	0.7396874558071848	0.6634226428820097
	annotation_met	0.699735262137758	0.25	0.7906443530468491	0.789214295844561

	annotation_rxn	0.716709543060149	0.285832415	0.807280819959982	0.7575646208501776
	annotation_gene	0.366666666666666	0	0.3666666666666667	0.3966360856269113
	annotation_sbo	0.272794093080752	0	0.5454545454545454	0.36363636363636365
RECON1	consistency	0.601671427971748	0.6017	0.6016714279717487	0.6016714279717487
	total_score	0.439489434651285	0.2731	0.5638214516442074	0.48677004184744743
	annotation_met	0.668088148294222	0.25	0.758997239203313	0.7571731413922304
	annotation_rxn	0.693501440494223	0.2786	0.781074578989575	0.7434657994000415
	annotation_gene	0.550323709536307	0	0.5503237095363079	0.5493088363954506
	annotation_sbo	0.181818181818181	0	0.45454545454545453	0.2727272727272727
Recon3D	consistency	0.815133084102353	0.815133084	0.8151330841023537	0.8151330841023537
	total_score	0.550241558590207	0.357006682	0.7165427920802498	0.6400238497110406
	annotation_met	0.557692607307003	0.25	0.7316429072213134	0.7304899898730233
	annotation_rxn	0.678556079664570	0.290141509	0.7498139412997904	0.7174606918238994
	annotation_gene	0.549896204033214	0	0.5498962040332147	0.5505634638196916
	annotation_sbo	0.272753001715265	0	0.6363636363636364	0.45454545454545453
STM_v1_0			0.90107716		0.9024426579144124
			0.389959025		0.635402112848361
			0.25		0.7873700938351327
			0.283791749		0.757061776904606
			0		0.39423026488329405
			0		0.36363636363636365
iSB619				0.9956	0.9955727472400974
				0.7512	0.6744846428802357
				0.802	0.7999306037473977
				0.8371	0.7819650067294751
				0.3667	0.39585352719439965
				0.5455	0.36363636363636365
iAF987				0.9921	
				0.7466	
				0.7844	
				0.8113	
				0.3667	
				0.5455	
